# Therapeutic impact of dietary ginger supplementation in chickens experimentally infected with coccidia—anti-oxidant, biochemical, and pathological evaluations

**DOI:** 10.3389/fvets.2025.1511759

**Published:** 2025-03-31

**Authors:** Alshimaa Saber, Amany Sayed Mawas, Atef M. Khalil, Ahmed I. Ahmed, Dina M. W. Shibat Elhamd, Esraa Ali, Ibrahim F. Rehan, Asmaa Elnagar, František Zigo, Mohammed Salah

**Affiliations:** ^1^Department of Pathology and Clinical Pathology, Faculty of Veterinary Medicine, South Valley University, Qena, Egypt; ^2^Division of Pathology and Clinical Pathology, Agricultural Research Centre (ARC), Animal Health Research Institute (AHRI), Qena, Egypt; ^3^Department of Poultry Diseases, Faculty of Veterinary Medicine, South Valley University, Qena, Egypt; ^4^Division of Clinical Poultry Diseases, Agricultural Research Centre (ARC), Animal Health Research Institute (AHRI), Qena, Egypt; ^5^Division of Parasitology, Agricultural Research Centre (ARC), Animal Health Research Institute (AHRI), Qena, Egypt; ^6^Department of Husbandry and Development of Animal Wealth, Faculty of Veterinary Medicine, Menoufia University, Shibin el Kom, Egypt; ^7^Department of Pathobiochemistry, Faculty of Pharmacy, Meijo University Yagotoyama, Nagoya, Japan; ^8^Department of Nutrition and Animal Husbandry, University of Veterinary Medicine and Pharmacy, Košice, Slovakia; ^9^Department of Biochemistry, Faculty of Veterinary Medicine, South Valley University, Qena, Egypt

**Keywords:** amelioration, coccidia, ginger (*Zingiber officinale*), immunity, histopathology

## Abstract

Coccidiosis is a significant disease with economic implications. It causes high mortality and morbidity, often associated with weight loss. This study investigated the effects of ginger supplementation on antioxidant status, biochemical parameters, and intestinal tissue histopathology in experimentally induced coccidiosis. One hundred one-day-old broiler chicks (Ross) were purchased from a local hatchery. Birds were kept in a clean, well ventilated and disinfected shed. The birds were given non-pelleted diets and water ad libitum throughout the period of the study (25 days). The aqueous ginger was administered orally to the chicks of the four dietary groups via drinking water at concentrations of 6 gm/L of water. All biosecurity measures were adopted according to standard protocol. The chicks were allocated into five groups: control negative (CN), control positive (CP) was infected with 3 × 10^4^ sporulated oocysts at day 14th of the experiment, the third group (GO) was given ginger only for 25 days, the fourth group (GB) was given ginger from day 4th to day 25th of the experiment, and the fifth group (GA) was given ginger from day 5th post infection to day 25th of the experiment. Ginger extract treatment reduced the fecal oocyst count in the infected group, particularly on day 10 post-infection. The alterations in differential leukocyte counts due to coccidial infection were ameliorated by ginger extract administration. Biochemically, compared to the infected group, ginger (*Zingiber officinale*) reversed the altered biochemical parameters (total protein, albumin, MDA, and SOD enzyme) associated with cecal coccidiosis. Immunologically, ginger extract treatment increased CD4 T cell counts and overexpression of INF-*γ* in the cecal epithelium. Histological examination revealed a significant reduction in goblet cell number and a decrease in the villus height (VH) to crypt depth (CD) ratio in infected chicks. Restoration of normal cecal histological structure and increased absorptive function and goblet cell number were observed in ginger-treated chicks compared to control-positive chicks. In conclusion, ginger supplements have effective therapeutic uses against intestinal coccidiosis as shown in the biochemical, immunohistochemical and histopathological results.

## 1 Introduction

Poultry sector is a primary source of white meat globally; however, the industry faces significant threats and challenges (1). Poultry coccidiosis is the most pathogenic protozoan disease affecting chickens, resulting in economic losses exceeding 3 billion dollars annually, thereby impeding the industry’s progress (2). Coccidiosis is economically significant due to its high infection and mortality rates in poultry (3). It infects the intestine and is caused by the protozoan parasite *Eimeria* (4). This parasite affects domestic animals, causing substantial epithelial cell damage, hemorrhagic diarrhea, poor growth rates, and reduced egg production (5). In poultry, avian coccidiosis, caused by intestinal *Eimeria* species, induces multiple responses in affected hosts (6). *Eimeria tenella* is highly prevalent in poultry, causing severe lesions and substantial losses, especially in young broilers and pullets (7). This species resides in the cecum and leads to serious harm, marked by intestinal hemorrhage, increased morbidity and mortality rates, weight loss, and emaciation (8). The infection induces immune responses (9), as well as hepatic (10), hematological (11), and leukocyte differential responses (12). However, pathological outcomes (13) and inflammatory responses (14) associated with *Eimeria* infection have not been thoroughly investigated.

*Eimeria* infection triggers antibody-dependent immune responses. In contrast, cell-dependent immune responses are activated by immune cells such as T-cells, NK cells and macrophages, which play roles in disease resistance (15). Epithelial and IEL cells are the initial defense against *Eimeria* infection. Inflammation in *Eimeria*-infected intestines is linked to macrophage invasion and T lymphocyte infiltration (16). CD4+ T cells, primarily T helper cells, are vital to the chicken’s immune response against coccidial infection (17). Three T lymphocyte subpopulations, Th1, Th2, and Th17, have been observed during coccidia infection in chickens (18–20), with each subpopulation aiding in pathogen defense (21). Th1 cells are crucial in the immune response against coccidial infection (20). CD4+ T cells, mainly Th1 and CD8+ T cells secrete IFN-*γ* during parasitic infestations (22). Both dendritic and macrophage cells release IL-12, which aids in Th1 cell differentiation during coccidial infection, supporting a robust cellular immune response through IFN-γ production (23, 24). IFN-γ transcription levels are upregulated in the cecal tonsils, intestinal IELs, and spleen during *E. tenella* infection (25).

Eimeriosis exacerbates oxidative stress in infected birds, evidenced by higher malondialdehyde (MDA) concentrations and reduced activity of antioxidant enzymes, including glutathione S-transferase, catalase, and superoxide dismutase, compared to uninfected birds (26). Consequently, various anticoccidial agents are used against coccidiosis, though many have reported side effects (27, 28). Numerous drugs are used to manage avian coccidiosis; however, extensive use has led to multidrug resistance and significant tissue residues (29). Current research focuses on medicinal products for their minimal toxicity and relatively lower production costs, highlighting the benefits of herbal treatments against coccidial infection (30).

Histopathologically, *Eimeria* spp. infection results in severe intestinal hemorrhage in the quail, with cecal swelling and softened intestinal contents. Marked histological deterioration is observed, characterized by significant intestinal damage and inflammation. Cecal tonsil cystic hypertrophy occurs following parasite dissemination, meront growth, and merozoite release (31). Consequently, global poultry production authorities actively seek safe, eco-friendly anticoccidial alternatives (32, 33). Several studies have demonstrated the efficacy of natural plant extracts for coccidiosis control (34). *Zingiber officinale* is recognized as a potent antioxidant; its aqueous and alcoholic extracts inhibit peroxidation (35). It contains active ingredients, including gingerol, which enhances digestibility and growth in chickens, and its derivatives are effective against coccidial infection (36–38). As using of the biochemical of coccidial drugs is noted expensive ad participating in the drug resistance. In a now a days using of hebal agents becomes very common, so that this study was designed to diminish the negative impact of these parasites on poultry, especially under small farmer’s conditions as well as elucidation the therapeutic and protective effects of ginger (*Zingiber officinale*) against avian coccidiosis.

## 2 Materials and methods

### 2.1 Materials

#### 2.1.1 Chicks

One hundred one-day-old chicks (Ross) brought from commercial hatchery for the experiment. They were housed in a clean experimental room with *ad libitum* access to feed and water. The diet provided was free of anti-coccidial drugs and met the essential nutritional needs of broilers Table 1. All standard management procedures and practices for commercial broiler production were followed. The chicks received oral vaccines for Newcastle disease, Gumboro, and infectious bronchitis.

#### 2.1.2 Ginger extract

Fresh ginger rhizomes were sourced from the market and carefully processed to obtain an active aqueous ginger extract (AGE).

#### 2.1.3 Parasites

Samples of cecal coccidiosis were collected from positively infected birds and examined in the Parasitology Division, Animal Health Research Institute, before preparation for infection.

### 2.2 Methods

#### 2.2.1 Ginger extract preparation

Ginger was washed, dried with gauze, cut into pieces, air-dried, and ground into powder. The powder was then stored in a sealed polyethylene bag. To prepare AGE, 14 grams of powdered ginger was mixed with 1 l of warm, boiled water. The mixture was incubated for 12 h at room temperature, cooled, and filtered through muslin cloth. The extract was stored at 4°C for subsequent use, with fresh extraction prepared weekly, following the procedures outlined in a prior report (39).

#### 2.2.2 Preparation of the parasite’s oocysts

The ceca from positive field cases were collected, and contents were harvested and placed in a 2.5% potassium dichromate (K_2_Cr_2_O_7_) solution at appropriate moisture and temperature in Petri dishes until sporulation. Mature sporulated oocysts were separated from fecal waste by a flotation centrifugation technique (40).

#### 2.2.3 Eimeria infection

Before experimental infection, all birds were screened to ensure they were infection-free. The purified and cleaned sporulated oocysts were prepared in distilled water and administered directly to the chicks, with each receiving approximately 3 × 10^4^ sporulated oocysts via a rubber syringe. Chicks were managed gently and housed individually, with clinical signs monitored up to the 10th day post-infection (41, 42).

#### 2.2.4 Experimental design

Table 2 at the end of the experiment, 10 birds per group were randomly selected. Before sacrifice, blood samples were collected from the wing vein for hematological and biochemical analysis. Selected birds were sacrificed, and the infection sites were examined for coccidial damage. Cecal tissues were harvested, with one portion frozen for oxidative stress, antioxidant, and quantitative PCR (qRT-PCR) analyses and another fixed in 10% buffered formalin for immunohistochemistry and histopathological examination.

### 2.3 Laboratory examinations

#### 2.3.1 Oocysts counting procedure

Fecal specimens were collected from all infected groups on days 6, 7, 8, 9, and 10 post-infection to assess oocyst count per gram of feces using the McMaster method, as described in Abd El Rahman et al. (43). Approximately 2 g of fresh feces was suspended and mixed in 58 mL of saturated sodium chloride solution, thoroughly mixed, and filtered through a sieve. A Pasteur pipette was used to transfer the purified solution into the McMaster chamber, where it was left for 5 min before counting.

#### 2.3.2 Hematological analysis

Blood samples were collected from the wing vein on day 10 post-infection and divided into two portions. One part was collected in EDTA-coated tubes (anticoagulant) for hematological analysis, specifically for total and differential leukocyte counts. White blood cells (WBCs) were counted using a Neubauer hemocytometer, with blood diluted using Natt-Herrick as a diluent following (44). Blood smears were prepared, fixed in methanol for 1 min, stained with Giemsa (Sigma Chemical Co., St. Louis, MO, USA) for 10 min, and rinsed twice in distilled water for 1 min each.

#### 2.3.3 Biochemical analysis

The second portion of blood was collected in plain tubes without anticoagulant for biochemical analysis, including plasma protein profiles [total proteins, albumin concentration, and albumin/globulin ratio (A/G ratio)]. Samples were allowed to clot for serum separation, centrifuged at 3000 rpm for 5 min, and stored at −20°C for later use. All biochemical kits used were sourced from Bio-Diagnostic Co., Dokki, Giza, Egypt, and protein profiles were measured colorimetrically as per (45, 46).

#### 2.3.4 Antioxidant analysis

Following autopsy, cecal tissue samples were collected aseptically, washed in PBS, and analyzed for oxidative stress and antioxidant activity. Malondialdehyde (MDA), purchased from Bio-Diagnostic Co., Dokki, Giza, Egypt (CAT. No. MD 25 28), was measured colorimetrically as outlined by Ohkawa et al. (47) and Dhindsa et al. (48). Superoxide dismutase (SOD) activity, purchased from Bio-Diagnostic Co. (CAT. no. SD 25 20), was measured colorimetrically following (49, 50).

#### 2.3.5 Immunohistochemistry analysis

Cecal tissue sections obtained from the autopsy were prepared for paraffin embedding, sectioned into 5-micron slices, and transferred onto slides using the avidin-biotin-peroxidase complex (ABC) method. Sections were incubated with rabbit polyclonal CD4 antibody (Novosbio, Cat. Nr.# NBP1-19371, Dil. 1:100), followed by the application of reagents (Vectastain ABC-HRP Kit, Vector Laboratories). Sections were stained using diaminobenzidine (DAB, Sigma) for antigen–antibody complex visualization, with negative controls in place. Immunohistochemistry results were evaluated by measuring the reaction area in 10 microscopic fields using Image J 1.53t (National Institutes of Health, USA).

#### 2.3.6 Analysis of mRNA of cecal interferon-gamma (INF-*γ*) by real-time PCR

Quantitative real-time PCR (qRT-PCR) was used to assess interferon-gamma (IFN-γ) mRNA levels in cecal tissue. Total cellular RNA was extracted with an RNeasy Mini Kit (Cat. no. 74104). qRT-PCR was performed using a 7,300 real-time PCR system (Applied Biosystems, Foster City, CA, USA), following the manufacturer’s instructions. The forward primer sequence was 5′-AAACAACCTTCCTGATGGCGT-3′, and the reverse primer was 5′-CTGGATTCTCAAGTCGTTCCATCG-3′. Gene expression changes were calculated using the cycle threshold (Ct) values and the 2^-ΔΔCt^ method, with reference to the standard gene (*sham*) (51).

#### 2.3.7 Gross lesion and lesion scoring

Lesions in intestinal tissues were evaluated 10 days after oocyst inoculation, following the method in Raman et al. (52). As shown in Table 3, lesions were scored from 0 to 4 based on macroscopic observations, including bleeding intensity and cecal wall thickness.

**Table 1:** Describe the feed compositions.

Composition	The ratio	
	Starter	Grower
Yellow corn	571	633
Soya bean oil	300	255
Bone meal	24.2	25.5
Corn glutin 60%	45	30
Sodium chloride	1.5	1.5
Fish meal	30	30
Mineral, vitamin mixture	3	2.7
L.D Methionine	1.2	1.2
L.Lysine	1.1	1.1
Total(Kg)	1,000	1,000
Calculated analysis		
Total protein%	23%	20%
Metabolizable	2,990	3,090

#### 2.3.8 Histopathological examination

On day 10 post-infection, cecal tissues were collected for histopathological analysis as described in Chand et al. (53). Approximately 1 cm of cecal tissue was preserved in 10% neutral buffered formalin, dehydrated through a graded alcohol series, and embedded in paraffin wax. The paraffin blocks were sectioned into 5 μm slices and stained with hematoxylin and eosin (H&E) (54). Additional staining was conducted using alcian blue (a histochemical method) and periodic acid–Schiff (PAS) for detailed histopathological assessment, specifically for epithelial alterations, villus height (VH) to crypt depth (CD) ratio, with the VH/CD ratio calculated by dividing VH by CD (55). Measurements of VH and CD in cm were performed using ImageJ software (National Institutes of Health, USA). Goblet cells were identified and counted based on the alcian blue stain, which colored them bluish (Alcianophilic reaction), while the PAS stain was used to examine the extracellular matrix (ECM). Five slides were analyzed per tissue block.

**Table 2:** Describes the groups and treatments.

Groups	Abbreviation	The description of treatment
Group 1	CN	negative control, Neither infected nor treated
Group 2	CP	Positive control, Infected with *E. tenella* at day 14th of the experiment but not treated with ginger.
Group 3	GO	Given aqueous ginger extract (6 g/L water) for 25 days.
Group 4	GB	Administered aqueous ginger extract (6 g/L water) from day 4 to day 25 of the experiment.
Group 4	GA	Infected and treated with aqueous ginger extract (6 g/L water) from day 5 Post infection to day 25 of the experiment.

**Table 3:** Lesion scoring degree.

Lesion degree	The description
0	The intestines show no macroscopic lesions.
+1	Few petechiae in the cecal wall with normal contents.
+2	Slight ballooning with thickening of the cecal wall and bloody content.
+3	Moderate swelling of the cecum with the presence of cecal core.
+4	Complete inflation and filling of the cecum with caseous cores.

#### 2.3.9 Statistical analysis

The data obtained were statistically analyzed using SAS software version 6.12 TS020. An ANOVA was conducted according to the general linear models (GLM) procedure in SAS. All results are presented as mean ± SD. For goblet cell counts and VH/CD measurements, a student-paired *t-*test was applied, with a significance level set at *p* < 0.01.

## 3 Results

### 3.1 Effect of ginger extract treatments on fecal oocyst output per day (OPD)

**Figure 1:**
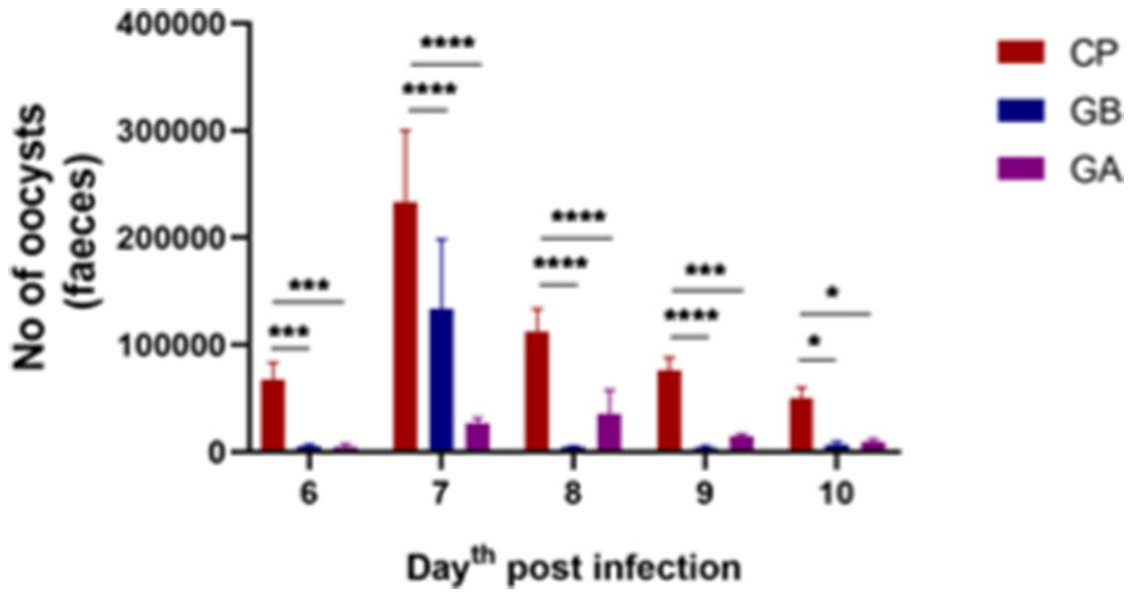
Oocyst output per day in *Eimeria* oocyst-inoculated groups from days 6 to 10 post-infection (pi). Infection with *Eimeria* oocysts increased the release of oocysts in the feces per day, peaking on day 7 post-infection, followed by a decrease in subsequent days. The count of oocysts was significantly reduced when infected birds were treated with ginger both before and after infection. Bars indicate means ± SE, with *N* = 10. A red asterisk indicates the difference between the control and other groups. *p < 0.05, **p < 0.01, ***p < 0.001, and ****p < 0.0001.

Fecal oocysts were counted from day 4 to day 10 post-infection (pi). Fecal oocyst production was first detected on day 6 pi, with no oocysts observed in the CN and GO groups. The oocyst-inoculated (CP) group exhibited the highest oocyst output per gram of fecal samples. Peak oocyst production was noted on day 7 pi, followed by a decline in subsequent days in all positive control birds. Oocyst production was significantly reduced in all ginger-treated birds compared to control-positive birds between days 6 and 10 pi (*p* = 0.001), as shown in Figure 1.

### 3.2 Effect of ginger extract treatments on total and differential leukocytic counts

**Figure 2:**
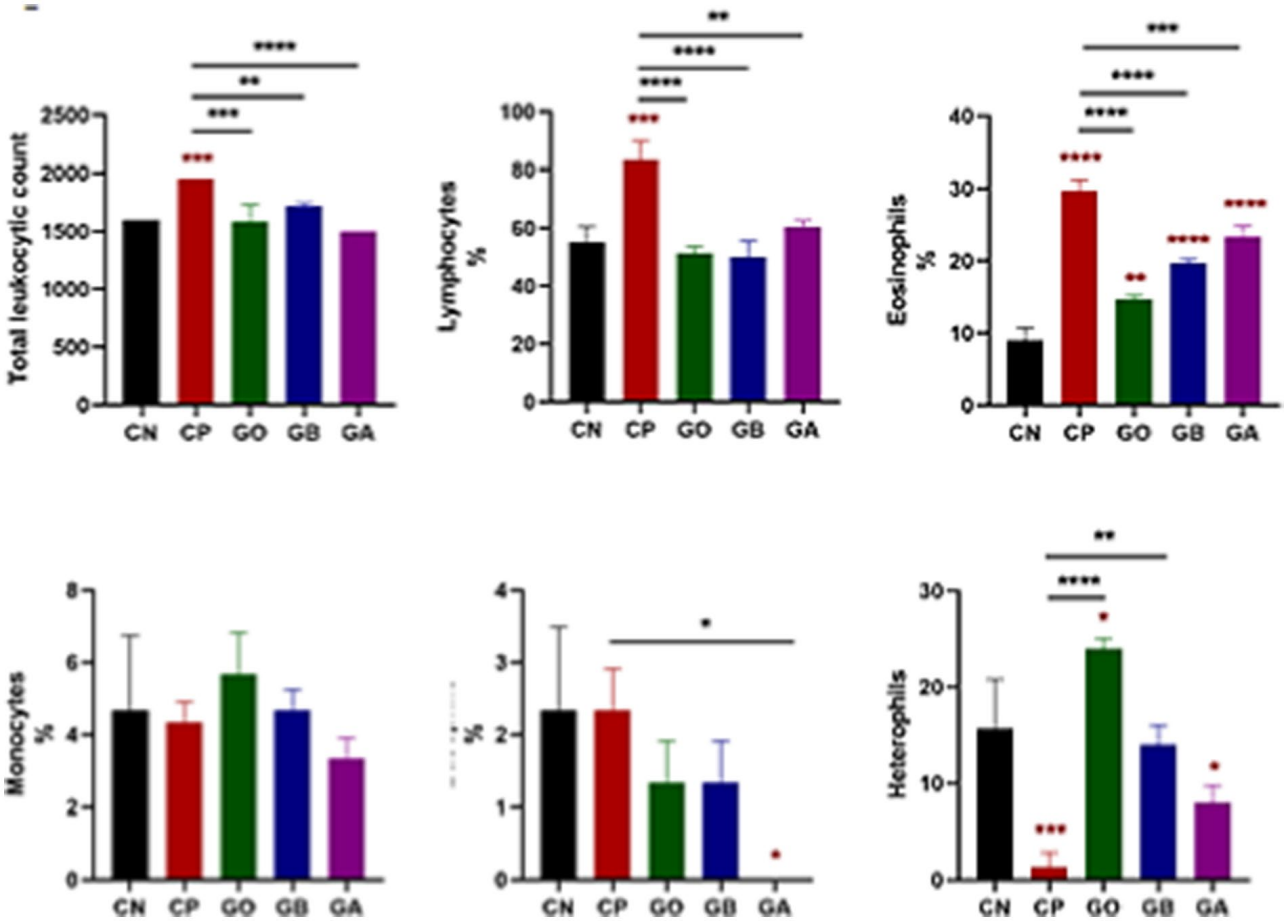
Impact of ginger extract treatment on the total and differential leukocyte count in infected and non-infected chickens. Infection with *Eimeria* oocysts significantly increased white blood cell (WBC) count, which decreased after ginger extract treatment. The infection exhibited a significant increase in the percentages of lymphocytes, eosinophils, and basophils, all significantly recovered following ginger extract treatment. The percentage of heterophils significantly decreased due to oocyst infection, but this percentage was corrected after ginger extract treatment. Bars indicate means ± SE, with *N* = 10. A red asterisk indicates the difference between the control and other groups. *p < 0.05, **p < 0.01, ***p < 0.001, and ****p < 0.0001.

Coccidial infection significantly elevated total leukocyte count in *Eimeria*-infected, non-treated (CP) birds compared to healthy birds. In other groups, leukocyte counts increased significantly but were not markedly higher than healthy birds (*p* = 0.001). Treatment with ginger extract, either before or after infection, significantly reduced leukocyte numbers. At the same time, a moderate increase was observed in ginger-only (GO)-treated, non-infected birds compared to control-positive birds (*p* = 0.0001), as shown in Figure 2. Regarding lymphocyte percentage, there was a significant increase in *Eimeria*-infected, non-treated birds compared to healthy birds (*p* = 0.001). In contrast, lymphocyte percentages were significantly reduced in the GO, GB, and GA groups compared to control-positive birds (*p* = 0.001). Eosinophil percentages increased in all infected groups (CP, GB, GA, and GO) compared to healthy birds (*p* = 0.0001). Ginger treatment reduced eosinophil percentages in infected birds compared to CP birds (*p* = 0.0001). In the GA group, ginger treatment post-infection significantly lowered basophil percentages compared to the CN and CP groups (*p* < 0.05). No significant changes in monocyte percentages were noted across all groups relative to the CN group. Heterophil counts were lower in *Eimeria*-infected (CP) birds compared to the CN group (*p* = 0.001), while heterophil percentages increased significantly in ginger-treated birds compared to the CN group (*p* < 0.05). In birds treated with ginger but free from infection throughout the experiment, heterophil counts increased compared to control-negative and positive birds (*p* = 0.05), as illustrated in Figure 2.

### 3.3 Effects of ginger extract treatments on biochemical analysis

**Figure 3:**
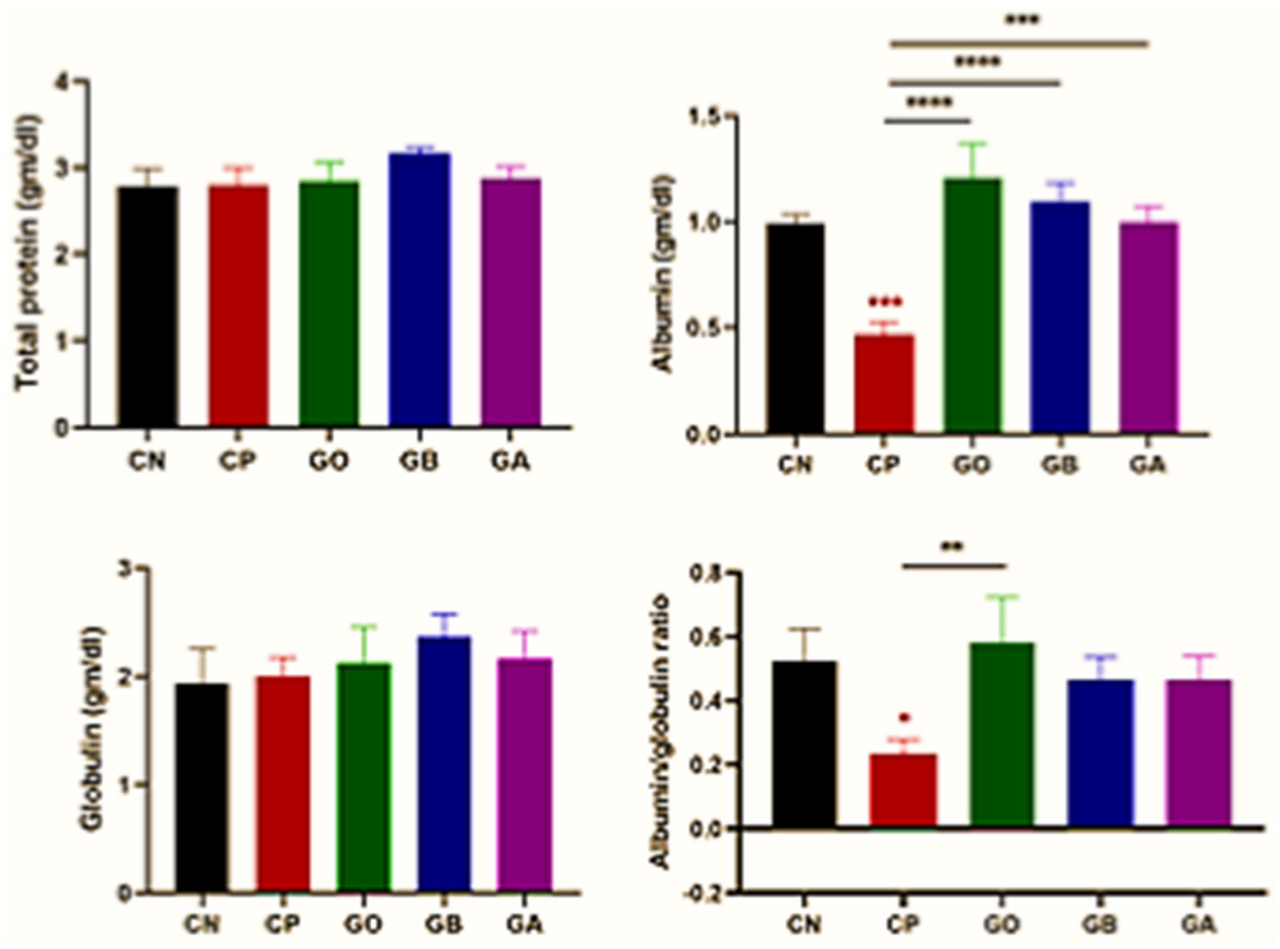
Impact of ginger extracts on the total protein, albumin, and globulin concentrations in infected and non-infected birds. No changes were observed in the total protein and globulin concentrations across all groups. However, albumin concentration and its ratio to globulin were decreased in the CP group and increased following ginger extract treatment. Bars indicate means ± SE, with *N* = 10. Red asterisks indicate the difference between the CP group and other groups. *p < 0.05, **p < 0.01, ***p < 0.001, and ****p < 0.0001.

*Eimeria* infection did not affect total protein and globulin levels (*p* > 0.05) across all exposed groups, including ginger-only treated (GO) birds, compared to CN birds. Albumin concentration was significantly reduced (*p* = 0.001) in CP birds compared to CN birds. A marked increase in albumin concentration (*p* = 0.0001) was observed in GO, GB, and GA birds compared to CP birds, with no significant changes in the other groups relative to CN birds. A reduced albumin-to-globulin (A/G) ratio (*p* < 0.05) was noted in CP birds versus CN birds, while this ratio increased in GO birds compared to CP birds (*p* = 0.01), with no significant differences observed in the other groups relative to control-negative and control-positive birds, as shown in Figure 3.

### 3.4 Effect of ginger extract treatments on oxidative products (MDA) and antioxidant enzymes (SOD)

**Figure 4:**
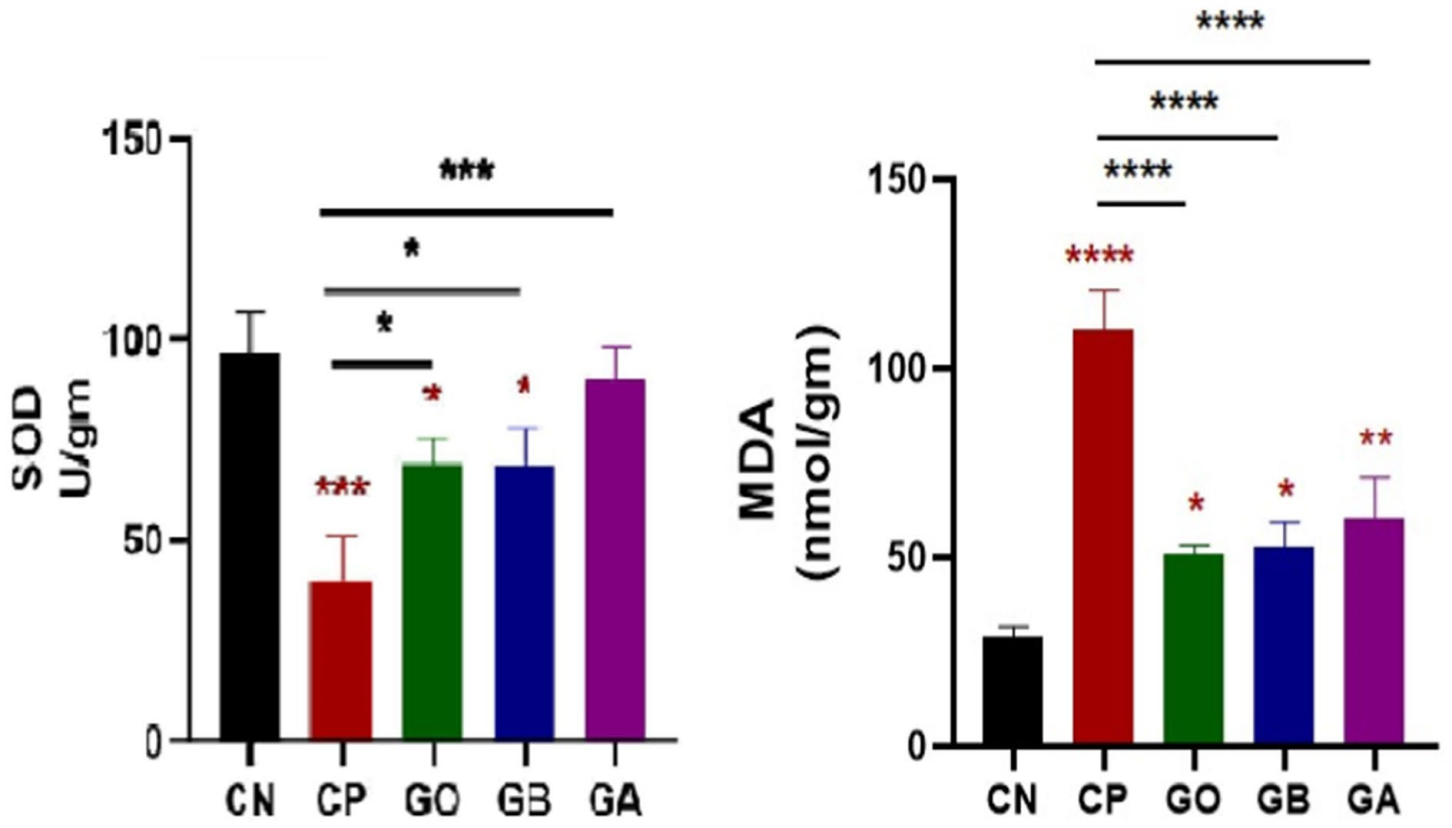
Impact of ginger extracts on SOD enzyme and lipid peroxidation (MDA) in cecal tissues of infected and non-infected birds. A decrease in SOD concentration was observed in infected birds compared to healthy birds. The concentration of MDA was elevated in the CP group compared to the CN group. Both SOD and MDA concentrations were normalized following ginger extract treatment. Bars indicate means ± SE, with *N* = 10. Red asterisks indicate the difference between the CP group and other groups. *p < 0.05, **p < 0.01, ***p < 0.001, and ****p < 0.0001.

Ginger extract demonstrated antioxidant effects, evidenced by significant changes in antioxidant enzyme activity (SOD) and tissue oxidation end-product levels (MDA). The data indicated a significant decrease in SOD activity in *Eimeria*-infected, ginger-non-treated, GO, and GB birds (*p* < 0.05) relative to healthy birds, with a marked increase in SOD activity in GO, GB, and particularly GA birds compared to CP birds (*p* = 0.001). A substantial increase in MDA concentration (*p* = 0.0001) was observed in CP, GO, GB, and GA birds relative to healthy birds, though MDA levels were significantly reduced in GO, GB, and GA groups compared to CP birds (*p* < 0.05), as shown in Figure 4.

### 3.5 Effect of ginger extract treatments on CD4^+^ T cells on the lamina propria of cecum

**Figure 5:**
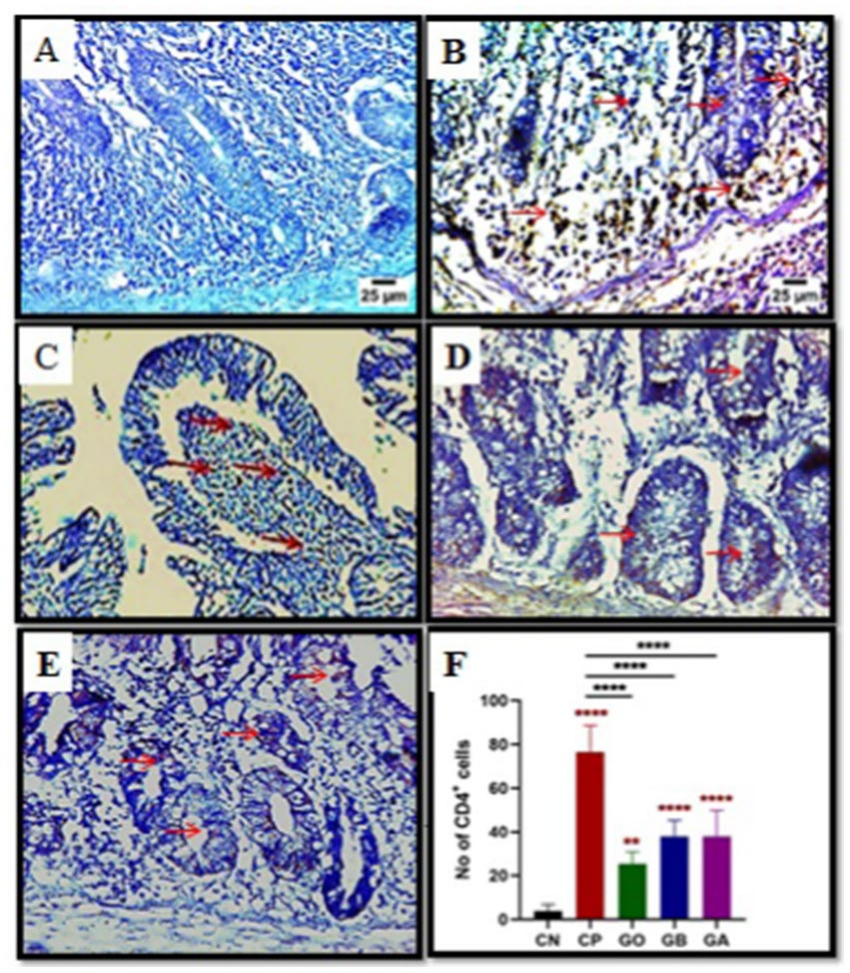
Impact of ginger extracts on CD4 T cell expression in cecal tissues of infected and non-infected birds. **(A)** No CD4 T cells were expressed in the control group. **(B)** Increased CD4 T cell expression (indicated by arrows) was observed in infected birds (CP) compared to healthy birds. **(C)** The ginger-only treated group exhibited mild CD4 T cell expression. The expression of CD4 T cells in ginger-treated birds before infection **(D)** and after infection **(E)** was lower than that in the positive control group (magnification = 400). **(F)** The impact of ginger extract on CD4 T cells activation bars indicate means ± SE, with *N* = 10. Red asterisks denote the difference between the CP group and other groups. **p < 0.01 and ****p < 0.0001.

CD4+ cell activation induced by cecal coccidiosis was assessed through immunohistochemical (IHC) examination. The data showed a significant increase (*p* = 0.0001) in CD4+ T cell expression in CP, GO, GB, and GA birds compared to CN birds. Additionally, a notable increase (*p* < 0.01) in CD4+ T cell expression was detected in CP birds compared to healthy birds. Conversely, CD4+ T cell proliferation decreased significantly (*p* = 0.0001) in GO, GB, and GA birds compared to CP chicks, as illustrated in Figure 5.

### 3.6 Effect of ginger extract treatments on interferon-gamma (INF-*γ*) down-regulation

**Figure 6:**
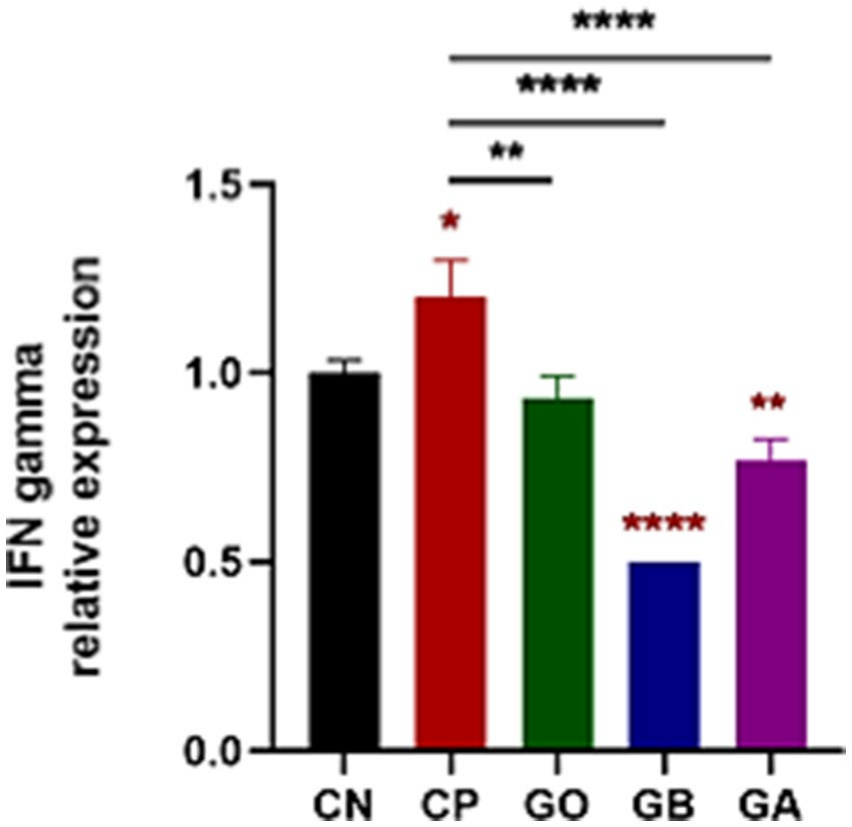
Impact of ginger extracts on IFN-*γ* expression in cecal tissues of infected and non-infected chickens. Increased IFN-γ expression was observed in infected birds compared to healthy birds. IFN-γ was highly expressed in infected chickens without ginger extract treatment. However, IFN-γ expression decreased in the GB and GA groups. Bars indicate means ± SE, with *N* = 10. Red asterisks mean the difference between CP and other groups. *p < 0.05, **p < 0.01, and ****p < 0.0001.

Cecal tissue sections from all experimental groups were analyzed via qRT-PCR for IFN-γ expression on day 10 pi. A significant increase in IFN-γ was observed in CP birds compared to CN birds (*p* < 0.05). Conversely, IFN-γ expression was markedly reduced in GB and GA birds (*p* = 0.0001) compared to healthy and CP birds. No significant difference was found in IFN-γ levels between GO birds and CP or CN birds (*p* = 0.01), as shown in Figure 6.

### 3.7 Histopathological effect of ginger before and post-infection

#### 3.7.1 Gross results and lesion scoring

**Figure 7:**
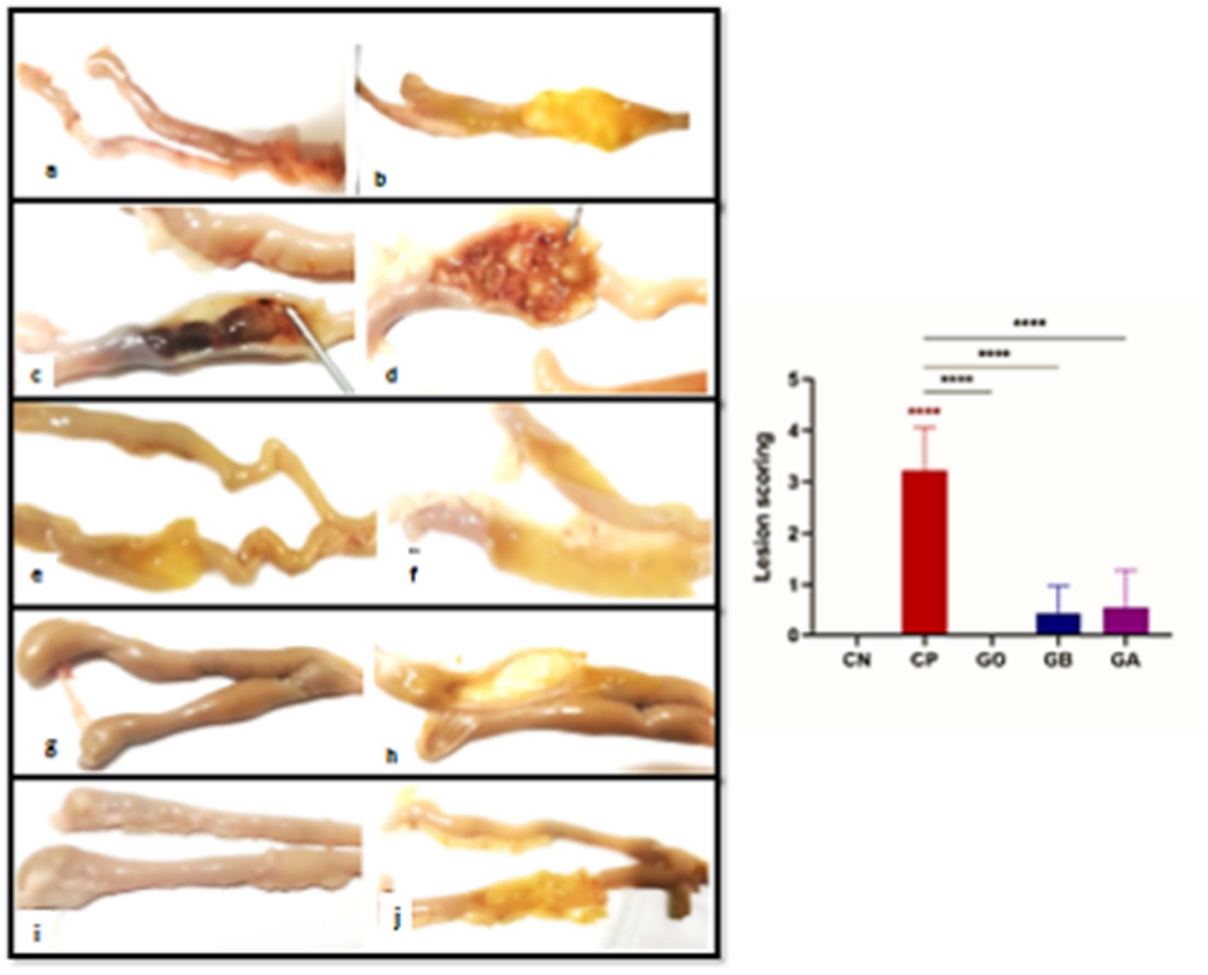
**(A–J)** Photographs of intestines from the CN group displaying an apparently normal intestinal wall **(A,B)**. The intestine of the infected CP group with cecal coccidiosis shows extensive ballooning and hemorrhagic changes **(C)**, along with a markedly thickened intestinal wall **(D)**. The intestine of the GO group exhibits a normal appearance of intestinal contents and mucosa **(E,F)**. The GB group’s intestine displays reduced ballooning **(G)** with a semi-thin wall and mucosa **(H)**. The intestine of the GA group shows minimal dilation **(G)** with pinpoint hemorrhagic foci **(H)**. Red asterisks mean the difference between CP and other groups. ****p < 0.0001.

Gross features were photographed on day 10 post-infection, revealing that the infected birds with coccidia exhibited severe cecal congestion, hemorrhage, complete inflation, and cecum distension, accompanied by caseous cores. A moderate to severe thickness of the cecum was observed in the positive control group. In contrast, the treated groups displayed mild to moderate petechial hemorrhages, with some birds appearing healthy. Mild thickness of the cecum was noted in the treated groups. No lesions or thickening were observed in the negative control groups (CN and GO) (Figure 7). Statistically, there was an extremely significant increase (*p* = 0.0001) in lesion scores in the cecal tissue of the CP group compared to the CN group. However, there was a non-significant decrease (*p* < 0.05) in lesion scores in the other groups compared to the healthy birds. Conversely, there was an extremely significant decrease (*p* = 0.0001) in the lesion scores of the GO, GB, and GA groups compared to the CP chicks (Figure 7).

#### 3.7.2 Histopathological examination

**Figure 8:**
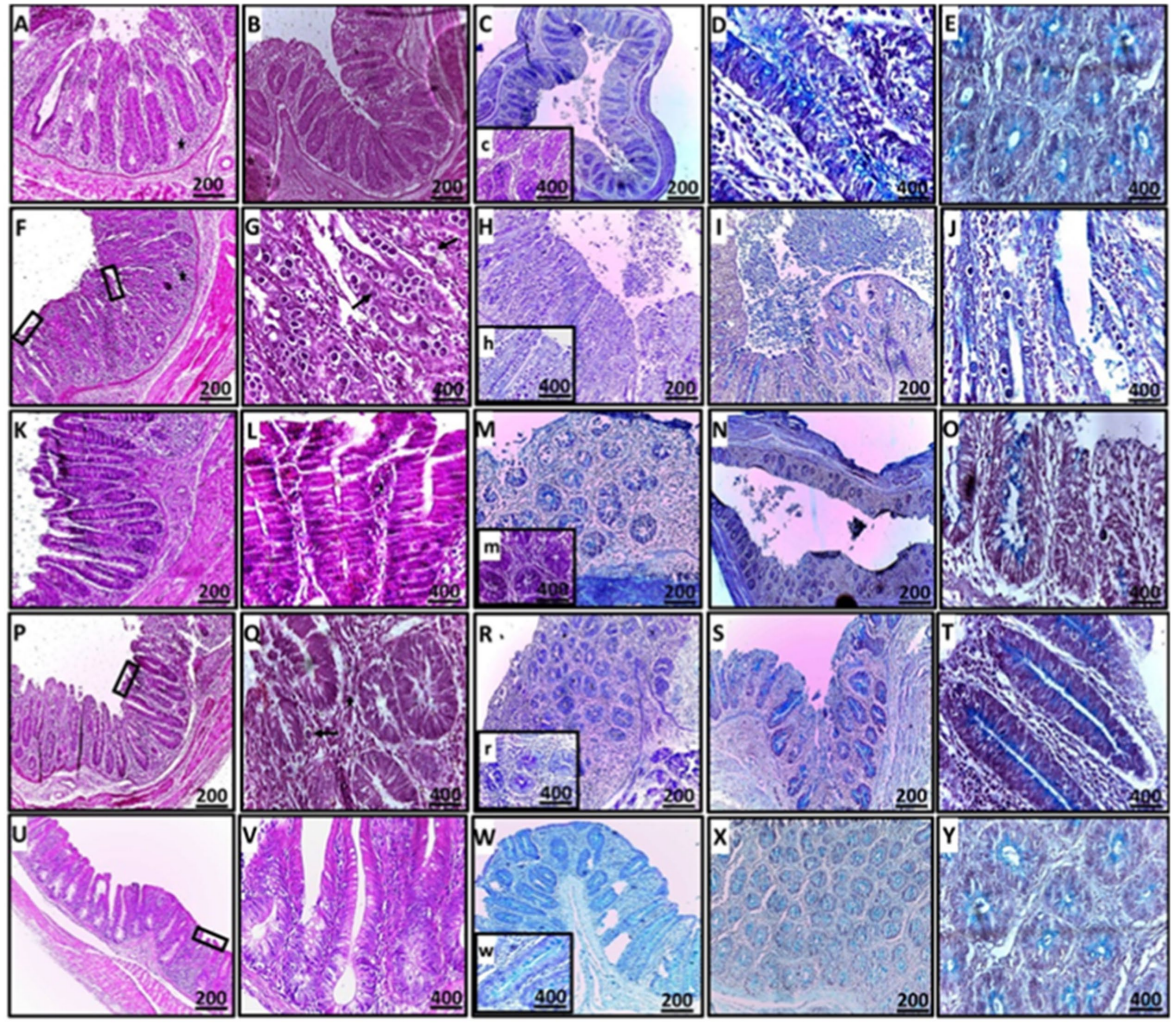
Histopathological sections of cecal tissues demonstrating mild erosions and ulcerations (rectangle), mild to no obvious epithelial coccidial stages (line arrow), and mild to moderate leukocytic infiltration (star) in the CN, GB, GA, and G groups compared to the CP group (**A–E**: CN; **F–J**: CP; **K–O**: GB; **P–T**: GA; **U–Y**: G). Staining methods include: (**A,B,F,G,K,L,P,Q,U,V**: H&E stain; **C,H,M,R,W**: PAS stain; **D,E,I,J,N,O,S,T,X,Y**: alcian blue stain).

**Figure 9:**
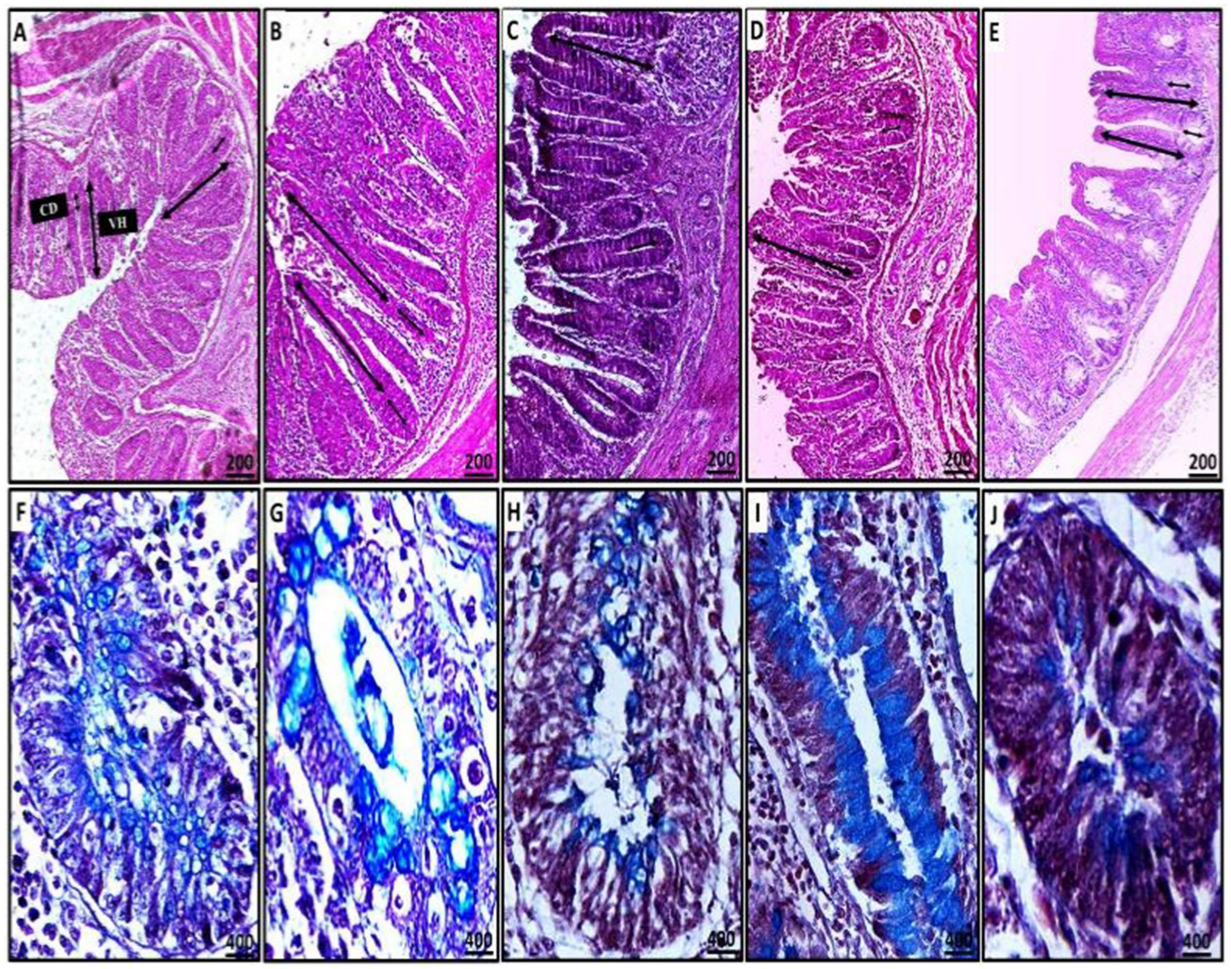
Representative microscopic images of cecal villous height (VH) and crypt depth (CD) (**A–D**: H&E stain) and the number of acid mucin-producing goblet cells (light blue) (**F–J**: alcian blue stain) (**A,F**: CN; **B,G**: CP; **C,H**: GO; **D,I**: GB; **E,J**: GA). A red asterisk indicates the difference between the control and other groups. *p < 0.05, **p < 0.01 and ****p < 0.0001.

Detailed histopathological alterations in the cecum were evaluated using H&E stains to assess the therapeutic effects of ginger supplements. Observations indicated distortion of the villus tip, disruption of the mucosal epithelium, and replacement by enterocyte cytoplasmic infiltration of coccidial stages, including schizonts and free merozoites (which appeared as small basophilic bodies surrounded by a halo), alongside inflammatory infiltration of mononuclear cells in the mucosal and submucosal layers (Figure 8). Ginger treatment significantly improved cecal function by restoring goblet cell activity and enhancing absorptive capacity through villus structure regeneration (Figure 9).

In the CN group, mild degenerative changes were noted, including intact villi, the absence of apparent stages of endogenous coccidia, and mild leukocytic expansion in the mucosal epithelium, reflecting normal histomorphology. In contrast, the CP group exhibited severe ulceration, villus atrophy, sloughing, and shortening of crypts, along with heavy infiltration of coccidial stages, including schizonts and free merozoites, which pose a risk of reinfection and cause significant damage to the intestinal tract. Heavy infiltration with inflammatory cells was observed, with lesions in the CP group resulting in the destruction and replacement of crypts.

For the treated groups (GB and GA), mucosal epithelial ulceration, luminal coccidial presence, and leukocytic infiltration were milder, with almost no visible coccidial stages, indicating near-normal histomorphology compared to the CP birds. The histopathological effects of ginger treatment in the treated groups are presented in Figure 8.

**Figure 10:**
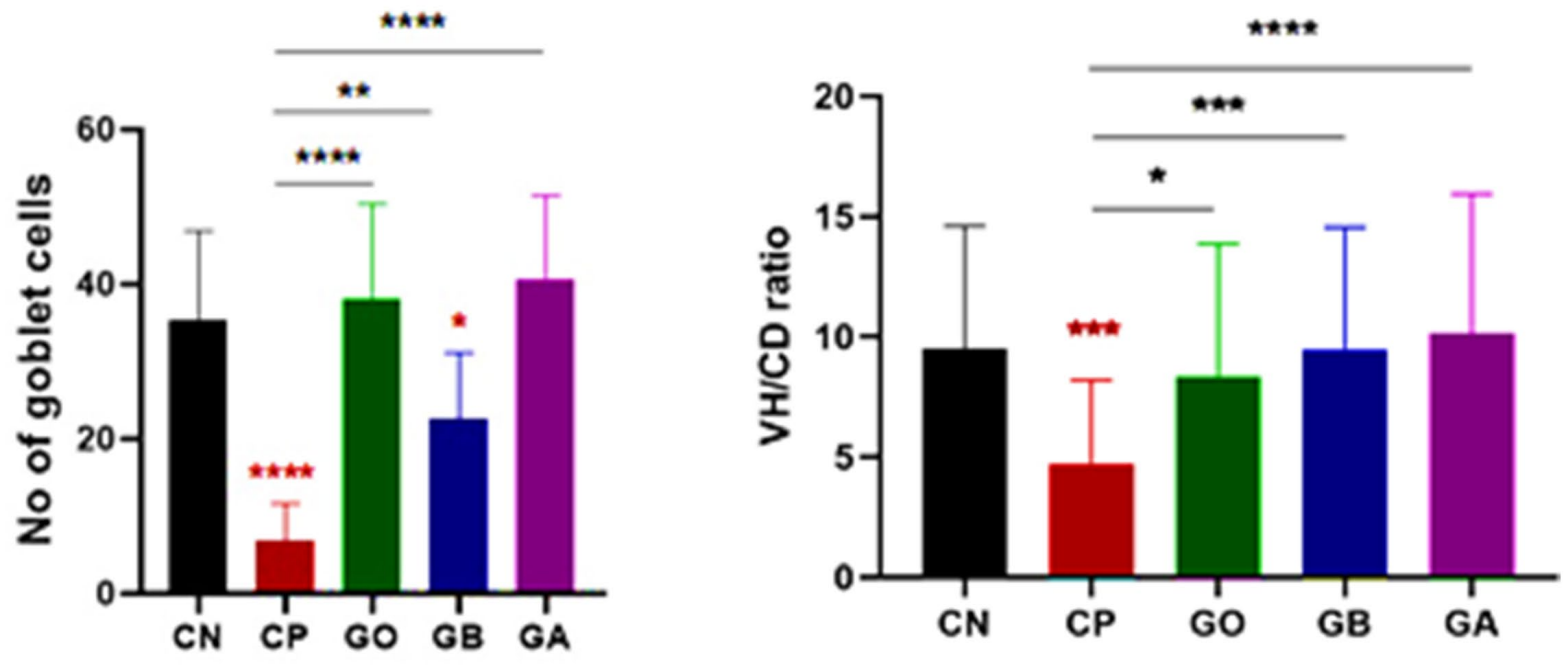
Statistical analysis of goblet cell numbers and the villus height/crypt depth ratio. Bars indicate means ± SE when *N* = 10. A red asterisk indicates the difference between the control and other groups. *p < 0.05, **p < 0.01, ***p < 0.001, and ****p < 0.0001.

The relationship between villus height (VH) and crypt depth (CD) was assessed, revealing an increase in the ratio of VH to CD. There was extreme statistical significance between the CN and CP groups (*p* = 0.0001), as well as between the CP and GB (*p* = 0.0001) and CP and GA (*p* = 0.0001) groups (Figure 10). No significant correlation was observed between ginger extract-treated birds and healthy birds (GB vs. GA, CN vs. GB, and CN vs. GA). Previous studies indicated that ginger meal activates the intestinal absorptive process. The number of goblet cells, an indicator of secretory function strength, was evaluated in various tissue sections across groups. Ginger supplementation as a feed additive significantly increased the number of goblet cells (Figure 9). A statistically significant reduction in the number of goblet cells was observed between the CN and CP groups (*p* = 0.0001), between the CN and GB groups (*p* = 0.05), and between the CP and GO groups. No changes were noted between the CN and GO groups or between the GA groups. Furthermore, there was a significant increase in goblet cell numbers in the GO (*p* = 0.0001), GB (*p* < 0.01), and GA (*p* = 0.0001) groups compared to the CP group (Figure 10).

### 3.8 Effect of ginger extract treatments on coccidial oocyst in tissue per villi

**Figure 11:**
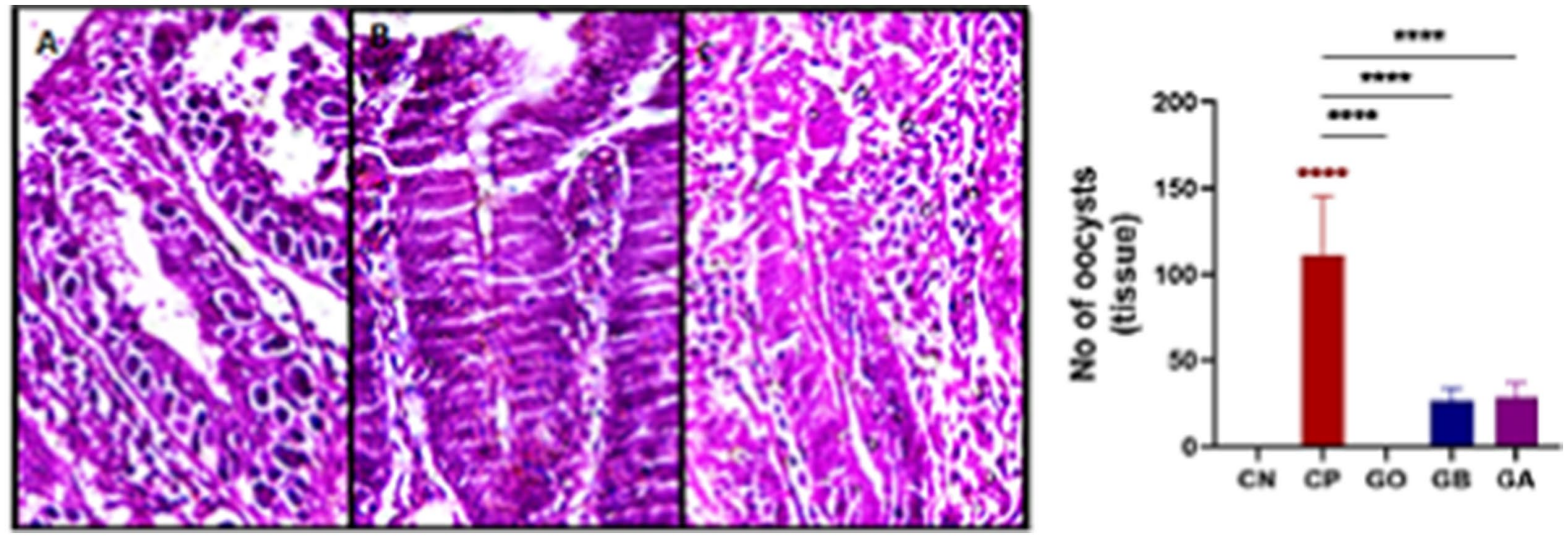
Photomicrograph showing the number of coccidial oocysts in the cecal villi (**A–C**: H&E stain) (**A**: CP, **B**: GB, **C**: GA). Red asterisks mean the difference between CP and other groups. ****p < 0.0001.

The number of oocysts in the cecal tissue of infected birds (CP) was elevated. However, following aqueous ginger extract treatment, the oocyst count significantly decreased in the GO, GB, and GA birds compared to the CP birds (*p* = 0.0001), as illustrated in Figure 11.

## 4 Discussion

This approach is especially relevant for diseases with economic impacts, such as coccidiosis, which causes gastrointestinal damage, reduces feed intake, and leads to lower body weight. This study was designed to evaluate the impact of ginger extract on intestinal function through antioxidant, biochemical, and histopathological assessments before and after experimental coccidial infection in broiler chickens. Eimeriosis is a disease induced by *Eimeria*, a genus of intestinal protozoa, which infects and proliferates in the epithelium lining of the digestive tract in birds, causing intestinal damage (inflammation, hemorrhage, diarrhea, etc.), morbidity, and mortality (56). Natural products offer a promising strategy to combat coccidiosis. Several commercially available herbal products have been shown to possess strong coccidiostatic and coccidicidal properties when added to the diets of chickens or other animals (57). Ginger, derived from the rhizomes of *Zingiber officinale*, has been used medicinally and as a spice (58).

In this study, fecal oocyst counts were conducted from day 6 post-infection. A high oocyst count was observed in the control-positive birds, persisting through day 10 post-infection. Oocyst production significantly decreased in all ginger extract-treated groups from day 6 to day 10 post-infection. These findings align with the results of Zidan et al. (59), who found ginger extract (*Zingiber officinale*) highly effective in treating pigeons with coccidiosis. Furthermore, Aljedaie and Al-Malki (60) noted that the reduction in oocyst numbers was attributed to ginger extract’s inhibitory and preventive effects against parasite invasion and cellular development within the avian intestinal tract, due to the bioactive components of Ginger, which has phenol derivatives that interacts with the parasite via an adsorption involving hydrogen bonding. These derivatives interact with proteins to form a phenol protein complex. This complex cause precipitation and protein denaturation leading to cell membranes lysis leading to reduction in the number of oocyst and the inflammation (61). Similar dysfunctions and limitations in parasitic stage development within the intestine have been observed with certain anticoccidial medications.

In the present study, a significant increase in lymphocyte numbers was observed in CP chicks compared to CN chicks, though no change was seen in the other ginger-administered groups. Conversely, a significant decrease was observed in lymphocyte percentages in the ginger-treated groups compared to CP birds. Lymphocyte elevation was induced by inflammatory changes in the cecum and intestines due to parasitic infection. Chronic infection stimulates lymphocyte proliferation and antigenic response, contributing to antibody formation and cell-mediated immunity (62).

Additionally, a significant increase (*p* < 0.05) in eosinophil counts was observed in all groups compared to CN birds, consistent with findings from Ahmed El-Shazly et al. (63) and Khaligh et al. (64).

Khaligh et al. (64), who reported heterophilia. However, reduced heterophil counts were observed in all treated groups compared to CP chicks. Eosinophilia in birds can increase in association with parasitism (intestinal parasites, migrating tissue parasites), as noted by Irizaary-Rovira (62). Furthermore, heterophil counts decreased in CP and GA chicks compared to CN chicks, while heterophil percentages increased in GO chicks compared to CN chicks. A significant increase in heterophil percentages was recorded in GO and GB chicks compared to the CP group. Heterophils, containing various types of granules, serve as the first line of defense against bacteria, protozoa, fungi, and certain viral infections (65). Treatment with ginger decreased the inflammation and necrosis in the cecal epithelium which intern decrease the infiltrated hetrophils around and increased in the circulation as mentioned by Zhang et al. (66) Monocytosis or heterophilia is a common response to acute and chronic inflammatory insults in animals and birds (67) because monocytes, macrophages, and dendritic cells, part of the hematopoietic system, play roles in protection and homeostasis maintenance.

In this study, total protein and globulin concentrations showed no significant changes in all exposed birds compared to CN and CP birds. These findings differ from Mohammad (67), who reported that hypoproteinemia, hypoalbuminemia, and hypoglobulinemia result from acute stress due to parasitic infection, leading to cortisol release and increased protein catabolism. Parasitic infection and tissue damage result in hemorrhage and plasma protein loss at the infection site, reducing the intestine’s protein and nutrient absorption capacity (68). A decrease in albumin concentration was observed in CP chicks compared to healthy chicks, likely due to nutrient malabsorption and hemorrhagic enteritis. Additionally, albumin levels increased in GO, GB, and GA chicks compared to CP chicks.

The immune system is highly specific, regulating antigen-dependent immune responses to control and inhibit pathogen development within the host. In all mammals, two main lymphocyte types, B lymphocytes (which produce surface immunoglobulins) and T lymphocytes (which function as T cell receptors), play central roles in adaptive immunity in birds (69). Immunohistochemical examination (IHC) revealed increased CD4+ T cells in GO, CP, GB, and GA compared to CN chicks. Comparatively, the CD4+ T cells in the GO, GB, and GA groups were significantly lower than those in the CP group due to the treatment by ginger which has anti proliferative effect in the CD4 T-cells and reduction of interferon-*γ* expression, which intern decrease the inflammatory events in the site of infection (70). Studies show that CD4+ helper T cells and CD8+ cytotoxic T cells are involved in immune responses against *Eimeria* infection (71, 72).

In this study, we observed a significant increase in interferon-γ expression in CP chicks compared to CN chicks, aligning with Dung (73), who reported that *Eimeria tenella* infection significantly raised Th1 cytokine gene expression (Interleukin (IL)-18 and IFN-γ). This is supported by Breed et al. (74), who demonstrated that IFN-γ is released in a specific cascade following the activation of circulating lymphocytes in *Eimeria*-infected birds. Although the previous authors Yun et al. (25) and Laurent et al. (75) observed IFN-γ secretion from lymphocytes isolated from the ceca of chickens infected with *E. tenella*, confirmed by mRNA expression in isolated lymphocytes, IFN-γ expression was lower in GO, GB, and GA chicks than in CP and CN chicks. This reduction was attributed to ginger extract’s anti-inflammatory properties due to its phenolic constituents, which decrease pro-inflammatory mediators like TNF and IL-6 (76, 77).

A decrease in SOD activity was observed in CP, GO, and GB birds compared to CN birds, consistent with Georgieva et al. (78) and Wang et al. (79), who found reduced SOD and catalase enzyme activities in infected versus healthy birds. Other researchers have reported SOD activity reduction due to oxidative stress and antioxidant imbalance following infection (80). Ginger extract treatment restored SOD activity in GO, GB, and GA chicks compared to CP chicks. Compared to healthy birds as described by Zhang et al. (66), MDA activity significantly increased in CP, GO, and GB. Our findings align with those of Eraslan et al. (81), who found that *E. tenella* oocysts increased lipid peroxidation in chickens, which was marked by elevated MDA substrate activity. This increase in MDA concentration during *Eimeria* infection (78, 82–84) is attributed to free radical overproduction, leading to lipid peroxidation. Elevated MDA levels during severe coccidial infection reflect high oxidative stress, while MDA activity significantly decreased in GO, GB, and GA compared to CP, confirming the immune-enhancing effects of ginger rhizome (10 g/kg) reported by Azhir et al. (85).

Coccidia infection causes severe cecal tissue damage, including various coccidial stages within enterocytes, with mononuclear cell infiltration, goblet cell reduction, and a lower villus height-to-crypt depth (VH/CD) ratio in infected groups (CP). In CN and ginger-treated groups (GB & GA), these histopathological changes were minimized, showing almost normal cecal tissue with improved function, characterized by the absence of coccidial stages and an increased goblet cell count and VH/CD ratio, indicating restored intestinal absorptive function. Ginger reduces coccidia’s number, viability, and infectivity in cecal tissue, confirming its immunologic and anti-parasitic effects against coccidiosis. Cecal tissue, commonly affected by *Eimeria* species, shows tissue distortion, destruction, and coccidial proliferation (86, 87). Active ginger components, such as gingerdoine and gingerol, promote digestion by stimulating digestive enzymes and speeding up food digestion (37, 88). Ginger’s amino acids and minerals enhance villus height, crypt depth, and intestinal epithelium regeneration, significantly increasing intestinal nutrient absorption capacity (36, 89). Intestinal function is linked to the VH/CD ratio; a lower VH/CD ratio decreases digestion, while a higher ratio improves it (90, 91). Goblet cells, which produce mucin, show heightened secretion when present in high intensity (92). Ginger repairs intestinal tissue by increasing goblet cell numbers, aiding defense through mucin production, and reducing inflammatory cell infiltration by lowering pro-inflammatory cytokines such as IL-1β, TNF-*α*, and IL-6 (93).

## 5 Conclusion

Utilizing ginger extracts (*Zingiber officinale*) before and during coccidial infection prevents the cellular development of oocysts through its immune-stimulating and antioxidant activities, as evidenced by the improvement in histopathological damage to the cecum. In conclusion, ginger, as a natural herbal remedy, exhibits significant protective and anthelmintic effects against coccidiosis.

## Data Availability

The original contributions presented in the study are included in the article/supplementary material, further inquiries can be directed to the corresponding authors.
